# Novel analytical method based on chemometric models applied to UV–Vis spectrophotometric data for simultaneous determination of Etoricoxib and Paracetamol in presence of Paracetamol impurities

**DOI:** 10.1186/s13065-023-01095-x

**Published:** 2023-12-07

**Authors:** Mona A. Abdel Rahman, Mohamed R. Elghobashy, Hala E. Zaazaa, Sally S. El-Mosallamy

**Affiliations:** 1 Analytical Chemistry Department, Faculty of Pharmacy, October 6 University, 6 October City, PO box 12858, Giza, Egypt; 2 Analytical Chemistry Department, Faculty of Pharmacy, Cairo University, Kasr El-Aini St, PO 11562, Cairo, Egypt

**Keywords:** Etoricoxib, Paracetamol, Impurities, PLS, ANN, MCR-ALS

## Abstract

The multivariate models that are used for spectral data analysis have many beneficial applications, and one of the important applications is the analysis of drugs and their impurities. Three Chemometrically-assisted spectrophotometric models have been proposed and validated. The proposed models are Partial Least Squares (PLS), Artificial Neural Networks (ANN), and Multivariate Curve Resolution-Alternating Least Squares (MCR-ALS). The advanced chemometric models were applied to resolve the significantly overlapping spectra of Etoricoxib (ETO) and Paracetamol (PCM), along with impurities of PCM namely; *P*-aminophenol (PAP) and *P*-hydroxy acetophenone (PHA). The proposed models succeeded in simultaneously analyzing the mixture of ETO and PCM along with the impurities of PCM. So, the proposed techniques can be used without requiring a separation step in the analysis of pharmaceutical formulation. Moreover, no significant differences were found when the results of the suggested and published chemometric models were compared statistically with the reported HPLC method.

## Introduction

The reactions of the body’s organs to the wound, and tissue damage are inflammation, pain, rash, osteoarthritis, and other illnesses [1]. The most common medications that are prescribed to treat chronic inflammation such as rheumatoid arthritis, and gout, and acute inflammation such as headache and postoperative pain conditions are nonsteroidal anti-inflammatory drugs (NSAIDs) [2].

**Fig. 1:**
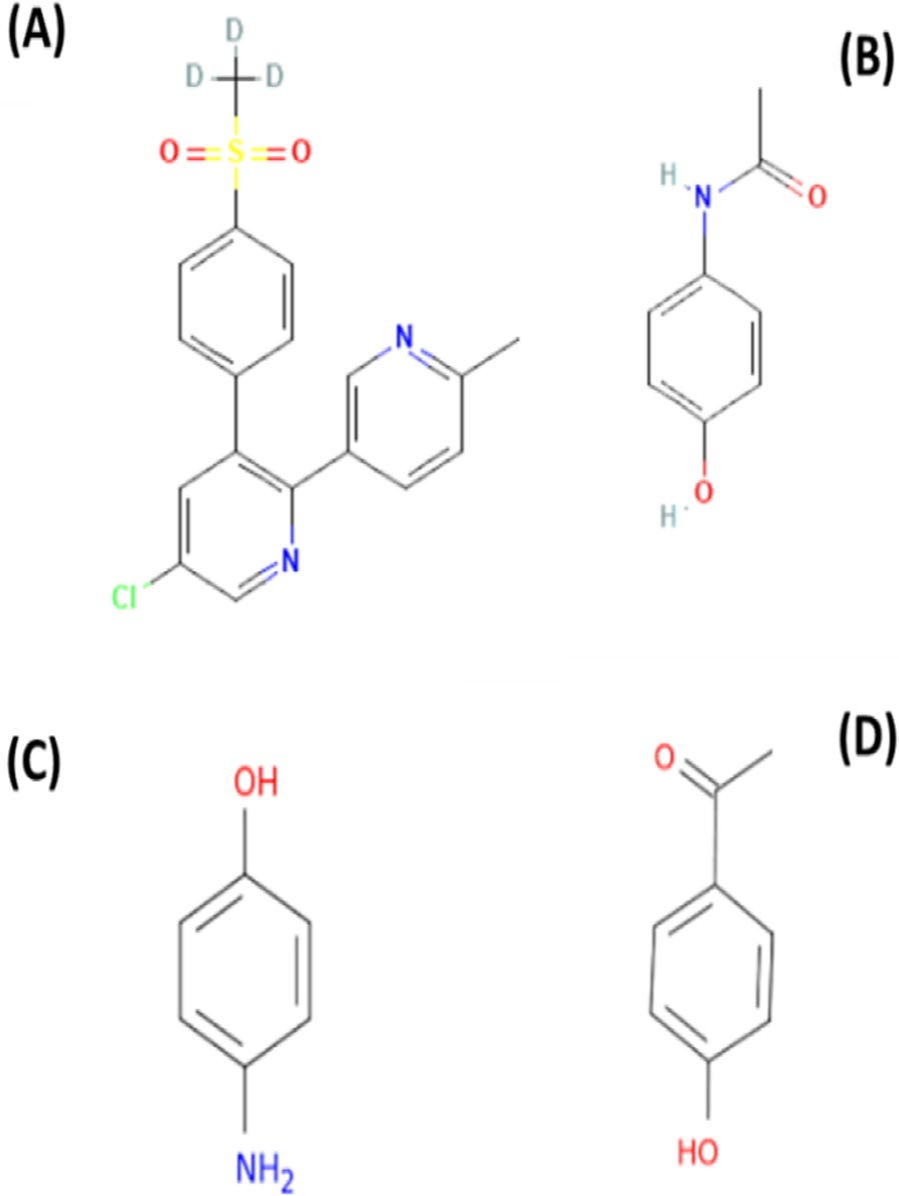
Structure of **a** Etoricoxib, **b** Paracetamol, **c** Para-aminophenol and **d** Para-hydroxy acetophenone

Etoricoxib (ETO) and Paracetamol (PCM) are new combinations frequently used as NSAIDs. Etoricoxib (ETO) is 5-chloro-3-(4-methanesulfonylphenyl)-2-(6-methylpyridin-3-yl) pyridine (Fig. 1a). ETO is a selective COX-2 inhibitor [3], used to decrease swelling and joint stiffness caused by osteoarthritis, rheumatoid arthritis, and gout [4], as well as to treat COVID-19 by overpowering a cytokine storm [5]. Paracetamol (PCM), N-(4-hydroxyphenyl) acetamide (Fig. 1b) considered a common antipyretic and analgesic drug [6], used in various pharmaceutical formulations to relieve pain and fever. It is approved in both British and United States pharmacopeias [7, 8], and it has been introduced as a supplementary treatment to reduce the fever brought on by COVID-19 infection [9]. Paracetamol is susceptible to degradation during storage like many other pharmaceutical compounds. In addition, during the manufacturing process, several impurities are produced. This makes it a challenging task to develop analytical techniques to evaluate active constituents in the presence of impurities.

P-aminophenol (PAP) (Fig. 1c) is a major PCM impurity, with nephrotoxic effects [10, 11] and teratogenic potential [12]. Figure 1d shows para-hydroxy acetophenone (PHA), a toxic impurity named in the British Pharmacopoeia as impurity E of PCM [7].

Several analytical techniques have been described in the literature to assay PCM such as HPLC [13–15], spectrophotometric [16–18], chemometric [19–21], and electrochemical analytical techniques [22, 23]. Furthermore, numerous techniques for determining ETO, including HPLC techniques, have been reported [24–26], HPTLC [27], spectrophotometric techniques [28], LC–MS/MS [29], and ion-selective [30]. A comprehensive literature review reveals only a few techniques for determining mixtures of ETO and PCM, such as HPLC [31–36], HPTLC [37], and spectrophotometric technique [38].

The reported HPLC method has some limitations, such as the time-consuming separation procedure and the reliance on toxic organic solvents like acetonitrile in the mobile phase. Additionally, suitable stationary and mobile phase selection for optimal peak resolution is a crucial parameter that requires fine-tuning. Alternatively, due to their simplicity of use and ability to beats the above-mentioned drawbacks, spectrophotometric methods are utilized as a powerful substitute for the analysis of the drugs. However, one of the challenges faced while analyzing multiple drugs simultaneously is undoubtedly spectral overlaps. So, one of the most potent tools for resolving this spectral overlapping problem is chemometrics. Chemometrics is the application of statistical and mathematical techniques used to build the most effective processes and to offer the most chemical knowledge via the analysis of data [39]. As a result, chemometrics has attracted much attention in recent years as an excellent processing technique for the spectral analysis of multicomponent mixtures in pharmaceuticals because of its ability to use multiple spectral intensities, which has a great impact on precision [40].

In the current study, several chemometric models, such as Partial Least Squares (PLS), Artificial Neural Networks (ANN), and Multivariate Curve Resolution-Alternating Least Squares (MCR-ALS), were applied to determine multicomponent mixture consist of PCM, and PCM impurities. To date, there is no chemometric models reported to resolve the spectra of both drugs together with the PCM impurities.

The aim of this work is to propose simple and smart chemometric models for the quantitative determination of ETO, and PCM in the presence of PCM impurities.

## Experimental

### Reagents and materials

Etoricoxib and Paracetamol, with purity of 99.5% and 99.94%, respectively, were provided by SIGMA Pharmaceutical Industries (Cairo, Egypt). The purity of the PAP and PHA that were obtained from Sigma-Aldrich was 99.73% and 99.61%, respectively. Methanol of HPLC grade was obtained from Sigma-Aldrich (Germany).

Pharmaceutical formulation: Intacoxia-P® tablets (Batch no: 5/UA/2017) obtained from Aagya Biotech Pvt Ltd (Manglaur Roorkee, Uttarakhand, India), labeled to contain 60 mg and 325 mg per tablet for ETO and PCM, respectively.

### Instrumentation

A Shimadzu UV–Visible dual-beam spectrophotometer, model UV-1800, equipped with a 1 cm quartz cell and UV-Probe 2.32 software was used to perform all spectrophotometric measurements (Shimadzu Scientific Instruments Inc., Kyoto, Japan). The PLS toolbox (version 2.1), ANN toolbox carried out in MATLAB® 8.1.0.604 (R2013a), and MCR-ALS toolbox [41] were used to implement all chemometric models.

### Standard solutions

An amount of ETO and PCM, equals to 15 mg and 20 mg, respectively, was transferred into two separate 100 mL volumetric flasks. After completing the volume of each flask to 100 mL with methanol, ETO and PCM concentrations were 150 μg mL^−1^ and 200 μg mL^−1^, respectively. Working solutions were prepared from stock solutions to reach final concentrations of 75 and 100 μg mL^−1^ for ETO and PCM, respectively. Ten mg of each PAP and PHA were accurately weighed into a volumetric flask (100 mL), and the volume was completed using methanol to give a final concentration of 100 μg mL^−1^.

### Procedure

#### Spectral characteristics

The absorption spectra of ETO, PCM, PAP, and PHA have been recorded over 200–400 nm utilizing methanol as blank. For further data analysis, the spectral data points with a wavelength range of 220–300 nm were imported into MATLAB®.

#### Construction of calibration and validation sets

The prediction performance of each calibration model was assessed using 18 samples as the calibration (training) set and 7 samples as the validation set. The calibration and validation sets' compositions contain various concentrations of ETO, PCM, PAP, and PHA ranging from 1.5–7.5, 2–10, 2–6, and 2–6 μg mL^−1^, respectively, as shown in Table 1. The solutions were prepared by mixing different volumes of each component from their respective working solutions in a 25 mL volumetric flask and then diluted them with methanol. PLS, ANN, and MCR-ALS are the multivariate calibration models used over the selected spectral range 220–300 nm with 0.1 nm intervals. Then we investigated and optimized all parameters of the models, before using them for simultaneous determinations of ETO and PCM, along with PCM impurities, in the validation set.

**Table 1:** Concentrations of ETO, PCM, PAP and PHA in the calibration and validation sets for the multivariate calibrations

Mix no	Concentration (μg mL^−1^)			
	ETO	PCM	PAP	PHA
1	4.5	6	4	4
2	4.5	2	3	2
3	1.5	4	2	6
4	3	2	6	6
5	1.5	10	6	4
6	7.5	10	4	3
7	7.5	6	3	6
8	4.5	4	6	3
*9*^a^	*3*	*10*	*3*	*5*
*10*^a^	*7.5*	*4*	*5*	*5*
*11*^a^	*3*	*8*	*5*	*4*
*12*^a^	*6*	*8*	*4*	*6*
13	6	6	6	5
*14*^a^	*4.5*	*10*	*5*	*6*
15	7.5	2	6	2
*16*^a^	*6*	*10*	*2*	*2*
17	7.5	2	2	4
18	1.5	2	4	5
19	1.5	6	5	2
20	4.5	8	2	5
21	6	2	5	3
22	1.5	8	3	3
23	6	4	3	4
*24*^a^	*3*	*6*	*2*	*3*
25	3	4	2	4

^a^The italic emphasis rows represent the validation set

#### Wavelength range selection

Various wavelength ranges were sought, but noisy and uninformative wavelength ranges were avoided in order to choose the best range for the proposed models that achieve higher selectivity and sensitivity.

#### Optimization of calibration regressions

For the PLS calibration model, mean centering algorithm as a preprocessing step and leave-one-out cross validation were adopted, and the root mean square error of cross-validation (RMSECV) was calculated to reach the optimal number of latent variables.

Artificial neural networks are computerized systems that mimic the way the human brain analyzes and processes data. A feed-forward model was trained to optimize the calibration model of ANN. We also tried to optimize the neuron^'^s number in the hidden layer as eight neurons were selected using the Purelin-to-Purelin transfer function. Additionally, the epochs number has been optimized.

In MCR-ALS calibration, applied constraints were the key parameter for optimization. A non-negativity constraint [non-negative least squares (nnl)] to both concentration and spectral profiles were used to reach the suitable parameters with the minimum number of iterations.

#### Assay of pharmaceutical formulation

The mean weight of ten tablets was determined, and finely powdered. An accurate weight equivalent to 18.5 mg of ETO and 100 mg of PCM from the crushed powder was weighed out, and dissolved in 50 mL methanol in a 100 mL volumetric flask. After sonicating for 15 min, methanol was added to adjust the volume, and solution was filtered to yield an initial stock solution claimed to contain 0.18 μg mL^−1^ ETO and 1.0 μg mL^−1^ PCM. For determination of ETO and PCM, the solution was further diluted where, 2 mL and 1 mL, respectively were transferred to two different 100 mL volumetric flasks from the previous filtrate and methanol was used to increase the volume to mark, resulting in a final concentration of 3.6 μg mL^−1^ ETO and 10 μg mL^−1^ PCM. Aliquots of the working solution were used for the quantification of ETO and PCM in the dosage form by the developed models.

## Results and discussion

Impurities arise through the synthesis process or from incorrect storage of drug products. PCM was prone to degradation and had impurities such as PAP and PHA. A few techniques only have been described for determining ETO/PCM in pharmaceutical formulations, but no one has described a chemometric models for the determination of ETO/PCM mixtures in the presence of PCM impurities. Therefore, it was of great importance to develop an accurate method for simultaneous determination of active ingredients and impurities that may be found in pharmaceutical dosage forms parallel advances in chemometrics, along with advances in analytical instrumentation and computational power, afford numerous beneficial tools that aid in the resolution and display of complex chemical information. Multivariate calibration models rely on the simultaneous inclusion of multiple spectral wavelengths and thus can resolve highly overlapping spectra. This provides better accuracy and precision than relying on a single wavelength.

Chemometric models can be used for the fast prediction of analyte concentrations using the multifactorial prediction analysis of the spectra of unknown samples. In quality control laboratories, multivariate calibrations are utilized for impurity profiling [42]. Additionally, chemometrics have several biomedical applications and used to generate metabolic profiling [43].

### Spectral characteristics and wavelength selection

**Fig. 2:**
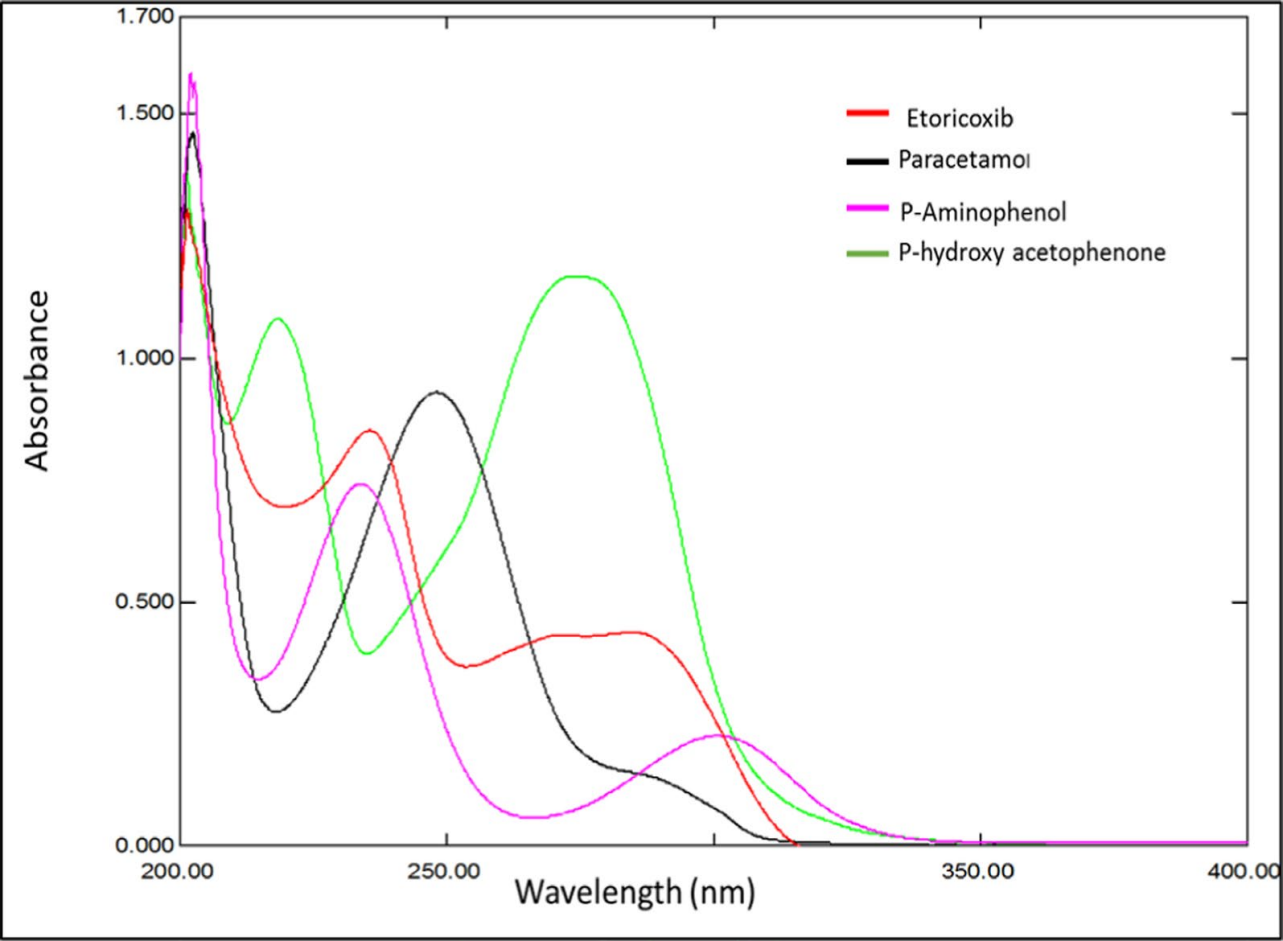
First-order absorption spectra of 10 µg mL^−1^ for ETO, PCM, PAP, and PHA using methanol as blank

The components’ UV spectra were recorded over the wavelength range of 200–400 nm. After a quick look at these spectra, a significant overlap was seen (Fig. 2). Using multivariate data analysis, we resolved the strongly overlapping spectra of the investigated active substance and the PCM impurity for the analysis of ETO, PCM, PAP, and PHA, three multivariate calibration techniques were developed. To obtain the best predictions, multivariate calibrations require a comprehensive experimental design to configure the calibration set.

The proposed models showed the best performance when the spectra were digitally scanned at 0.1 nm within the selected range of 220–300 nm. The other wavelengths were ignored due to noise that appeared within the range of 200–210 nm and poor absorption within the range of 300–400 nm.

### Construction of the models

A set of 25 laboratory prepared mixtures of the components under study includes calibration, and validation sets with concentration levels ranging from 1.5–7.5 μg mL^−1^ for ETO, 2–10 μg mL^−1^ for PCM, and 2–6 μg mL^−1^ for PAP and PHA were constructed using the four-factor five level design [44], where 18 samples serve as the calibrations set and the remaining 7 samples applied as a validation set (Table 1).

### Partial least squares (PLS)

**Fig. 3:**
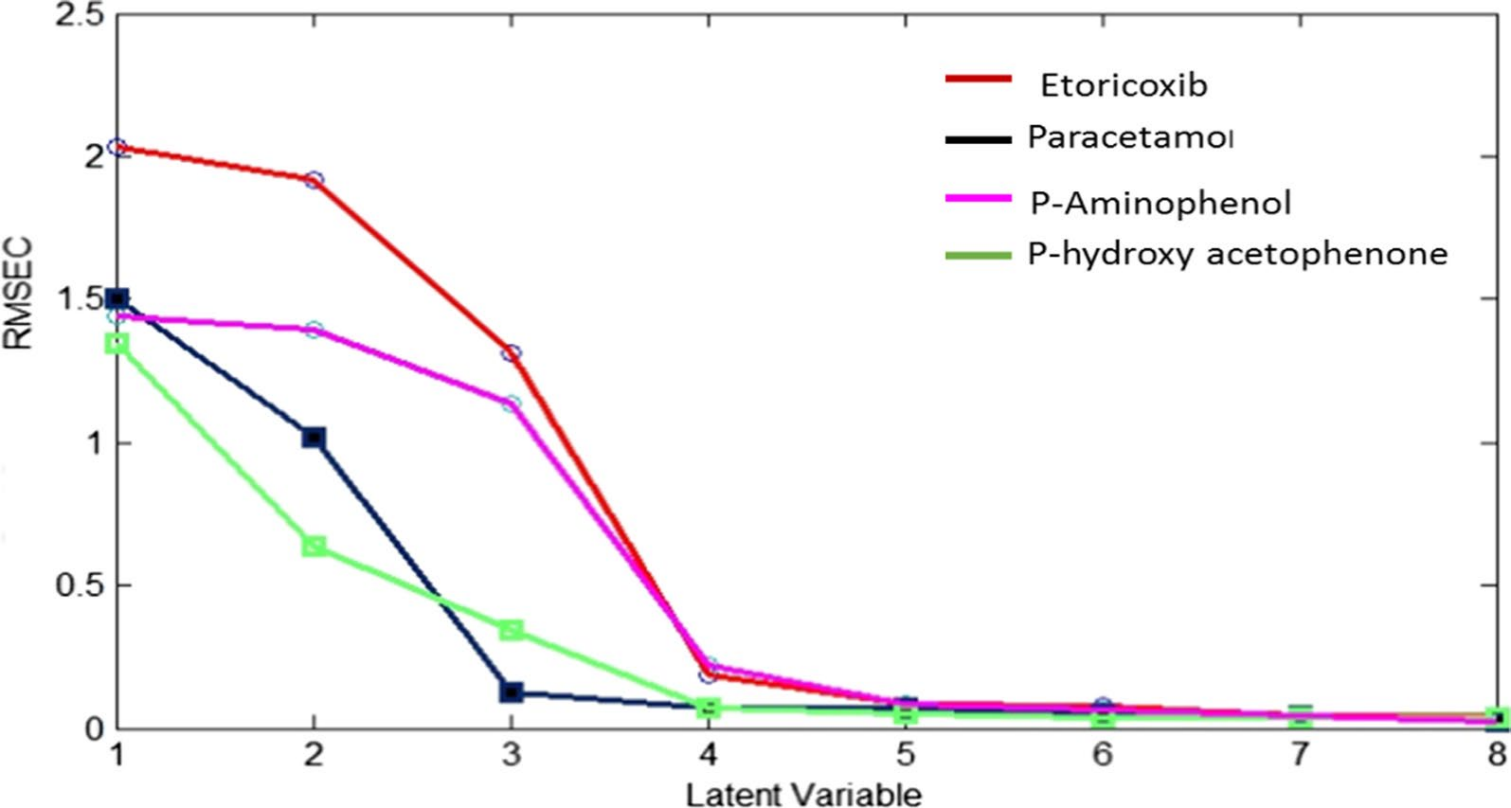
RMSEC plot of the cross validation results of the calibration set as a function of the number of latent variables used to PLS calibration

In quantitative analysis, PLS models are frequently utilized to obtain specific data from the spectrum of unselective data [45]. The PLS model, usually applied as a regression model to the spectral matrix of the calibration data to translate it into new spaces' dimensions known as latent variables (LVs). It was necessary to prudently determination of the optimal number of LVs to prevent losing important information and any overfitting of the model owing to insufficient or excess LVs. Therefore, leave-one-out cross validation method was utilized to reach the optimal number of the LVs, the calibration spectra remaining were modeled, and the root mean square error of calibration (RMSEC) was recalculated after the gradual addition of various LVs to the model according to criteria of Haaland and Thomas [46]. Before building the models, the data were either used as raw data or pre-processed using auto scaling or mean centering algorithms. Mean centering was the best pre-processing algorithm displaying good recoveries, RMSE and RSD. For all components his study, the optimal number of latent variables was revealed to be 6, as displayed in Fig. 3.

### Artificial neural networks (ANN)

Artificial neural networks operate via a network of structures based on neurons. The layers of the neurons are input, hidden, and output. A feed-forward networks are the networks used in this study. After the input layer receives the data, weights are created based on the input values and then transformed through transfer functions into output values. Network learning is accomplished by backpropagation. The estimates produced by the networks are then compared to the desired outputs. Errors are then calculated and returned backward via the network. This process of learning will continue until the networks are trained properly [47].

Through a trial-and-error method, various parameters for the networks were adjusted; to achieve the highest predicting abilities for them. These parameters implicate the number of neurons in the hidden layer, training functions, and transfer function pairs. The choice of the transfer function based on the characteristics of the analyzed data.

In this work, the Purelin–Purelin transfer function was appropriate for all analytes as predicted with the linear relationship between absorbance and concentration of analytes under investigation. The networks had been trained on a variety of training functions, it was found that there is no difference between them regarding RMSEP. As a training function, the TRAINLM-Levenberg–Marquardt backpropagation (TRAINLM) was preferred and selected to save time. Matching to the number of spectrum data points utilized, 801 neurons were applied in input layer, and 4 neurons were applied as an output layer, corresponding to the number of components that were computed to be determined in each sample. Several numbers of hidden neurons were examined to adopt the ideal number of neurons that enhanced the ANN's ability for prediction, 8 hidden neurons, and 500 epochs were found to be optimal.

**Fig. 4:**
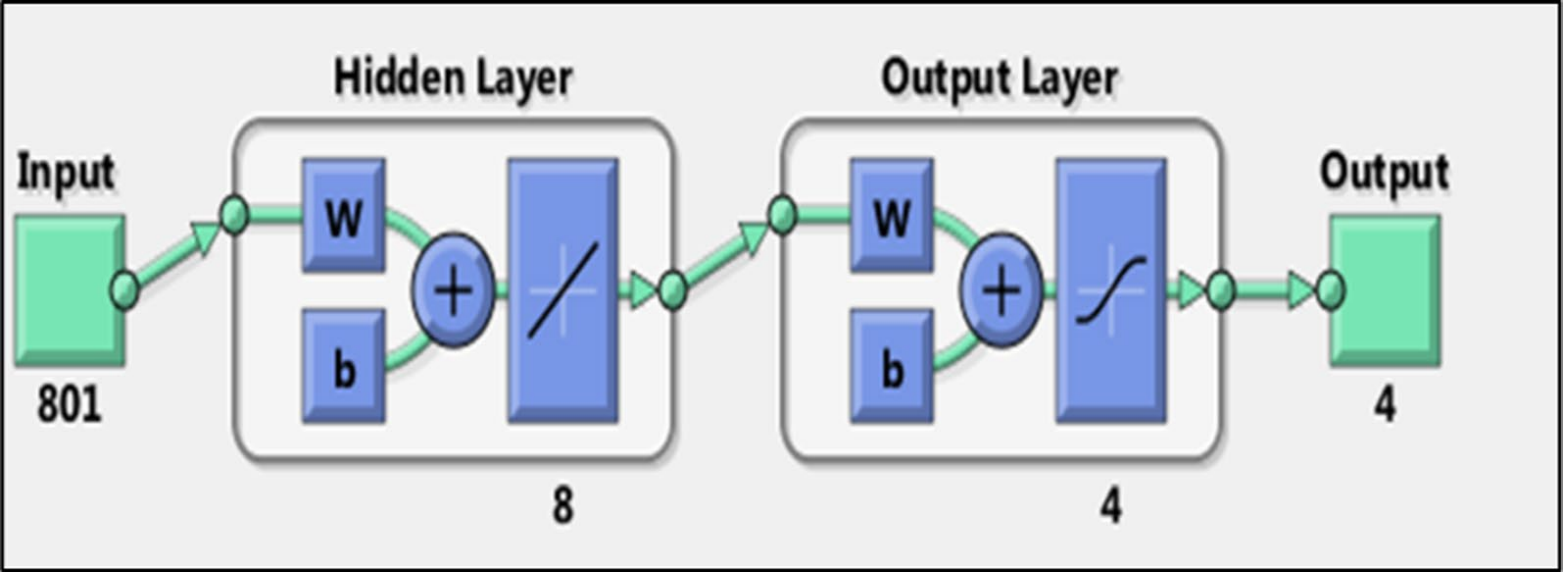
ANN Architecture for the prediction of the concentrations of the four component using different layer

**Fig. 5:**
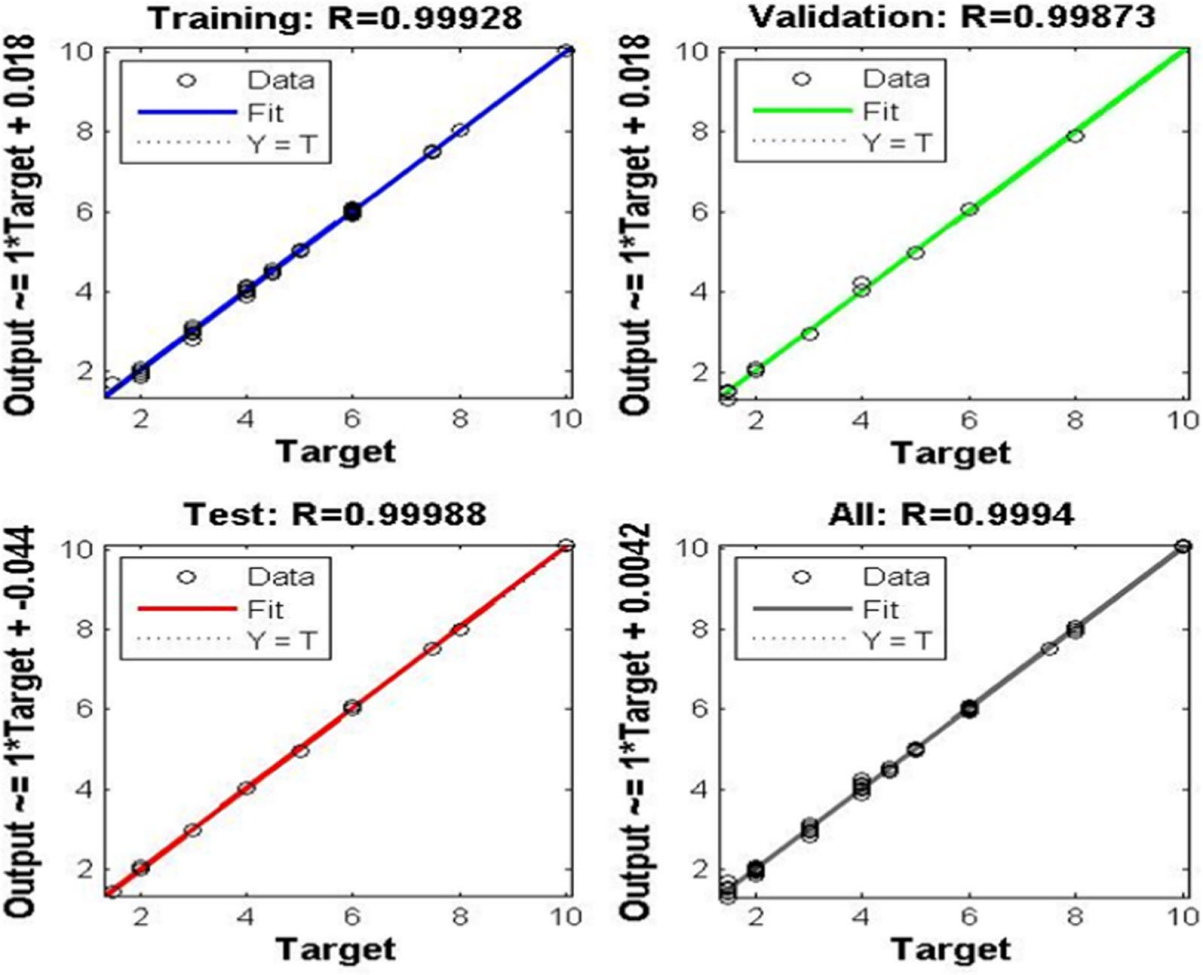
ANN diagrams of prediction for the training, test and validation

The ANN architecture revealed different layers applied to predict the concentration of the four components (Fig. 4). Figure 5 shows prediction diagrams for the training, and validation series of the chosen layers and neurons, r close to 1 for the training, and validation sets.

### Multivariate curve resolution-alternating least squares (MCR-ALS)

The key aim of MCR is to obtain pure response profiles of unresolved mixed constituents when no previous knowledge is accessible**.** It works by applying a bilinear model to break down the data matrix**.** At first, the initial estimation of the compounds, then followed by ALS optimizations of specific constraints applied to concentrations of the components and spectra profiles. The non-negativity constrains were applied to the concentration and spectral profiles, as well as to correlation constraints in concentration profiles [48]. The requirement for non-negativity constraints the concentration and spectra exist equal to or higher than zero. The optimization process of ALS was finished when a specific convergence criterion reached 20%. Convergence is often terminated when there are relative differences in the SD of residuals between ALS results and experimental data is smaller than a threshold value in two successive iterative procedures (often set to 0.1%).

In this study, evolving factor analysis was used to obtain a preliminary assessment with a log eigenvalue of − 2, this resulted in the creation of a five-factor model. Iterations continue until an ideal solution was found that satisfies both the predefined convergence criteria and the hypothesized limitations. The convergence ended up occurring after 10. The computed variance percentages (R^2^) and lack of fit (% lof) were 0.66154 and 99.9954, respectively, which were sufficient to support the goodness of the suggested MCR-ALS model.

**Fig. 6:**
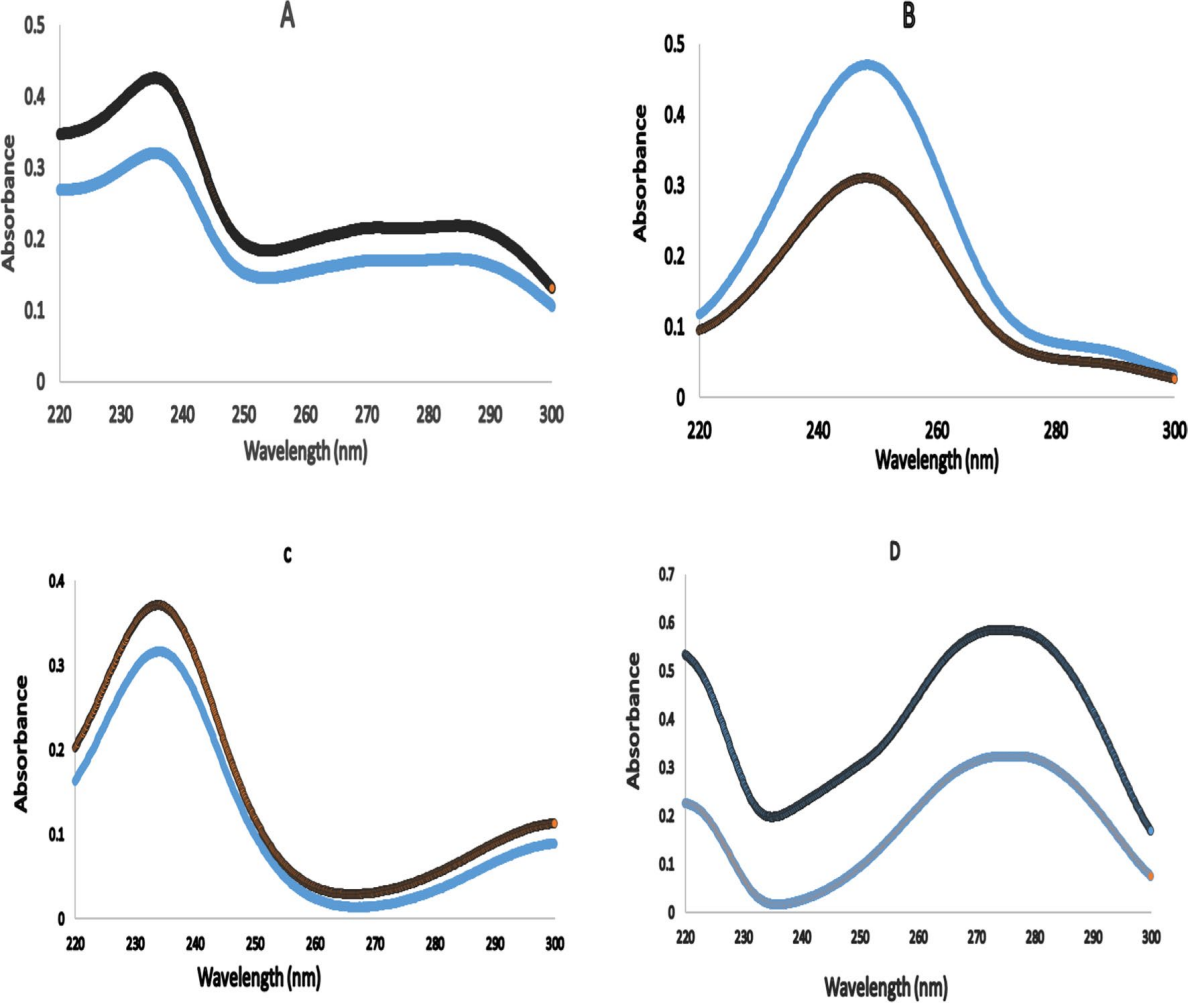
Original spectra ( ) and estimated spectra ( ) by MCR-ALS of **a** ETO, **b** PCM, **c** PAP, **d** PHA

The MCR-ALS model was used to estimate the spectrum profiles of the drugs and PCM impurities, as it provides qualitative significance in their algorithms. We observe that the estimated spectrum is similar to the original spectrum for each component (Fig. 6). The MCR-ALS model has the advantage of qualitative detection of components in addition to the ability of quantitative determination.

We constructed the three models to determine each analyte's concentration in the calibration sets, then calculated the correlation coefficient (r) and root mean square error of calibration (RMSEC), and good results were obtained as shown in Table 2.

**Table 2:** Performance parameters of the calibration calculated for each proposed model

Parameter	PLS				ANN				MCR-ALS			
	ETO	PCM	PAP	PHA	ETO	PCM	PAP	PHA	ETO	PCM	PAP	PHA
Slope^a^	0.9996	0.9997	0.9991	0.9993	0.9937	1.0012	0.09961	1.0103	1	1	1	1
Intercept^a^	0.0016	0.0015	0.0037	0.0028	0.03	− 0.0088	0.0054	− 0.0375	1.4787 × 10^–17^	− 2.334 × 10^–16^	− 2.3734 × 10^–16^	0
Correlation Coefficient (r)^a^	0.9996	0.9997	0.9991	0.9993	0.9995	1.00	0.9992	0.9982	0.9992	0.9996	0.9995	0.9996
RMSEC^b^	0.0430	0.0458	0.0446	0.0359	0.0550	0.0178	0.0439	0.0590	0.0224	0.0403	0.0424	0.0304

^a^Data of the straight line plotted between predicted concentrations of each component versus actual concentrations of calibration set

^b^Root Mean Square Error of Calibration

### Validation of the models

**Fig. 7:**
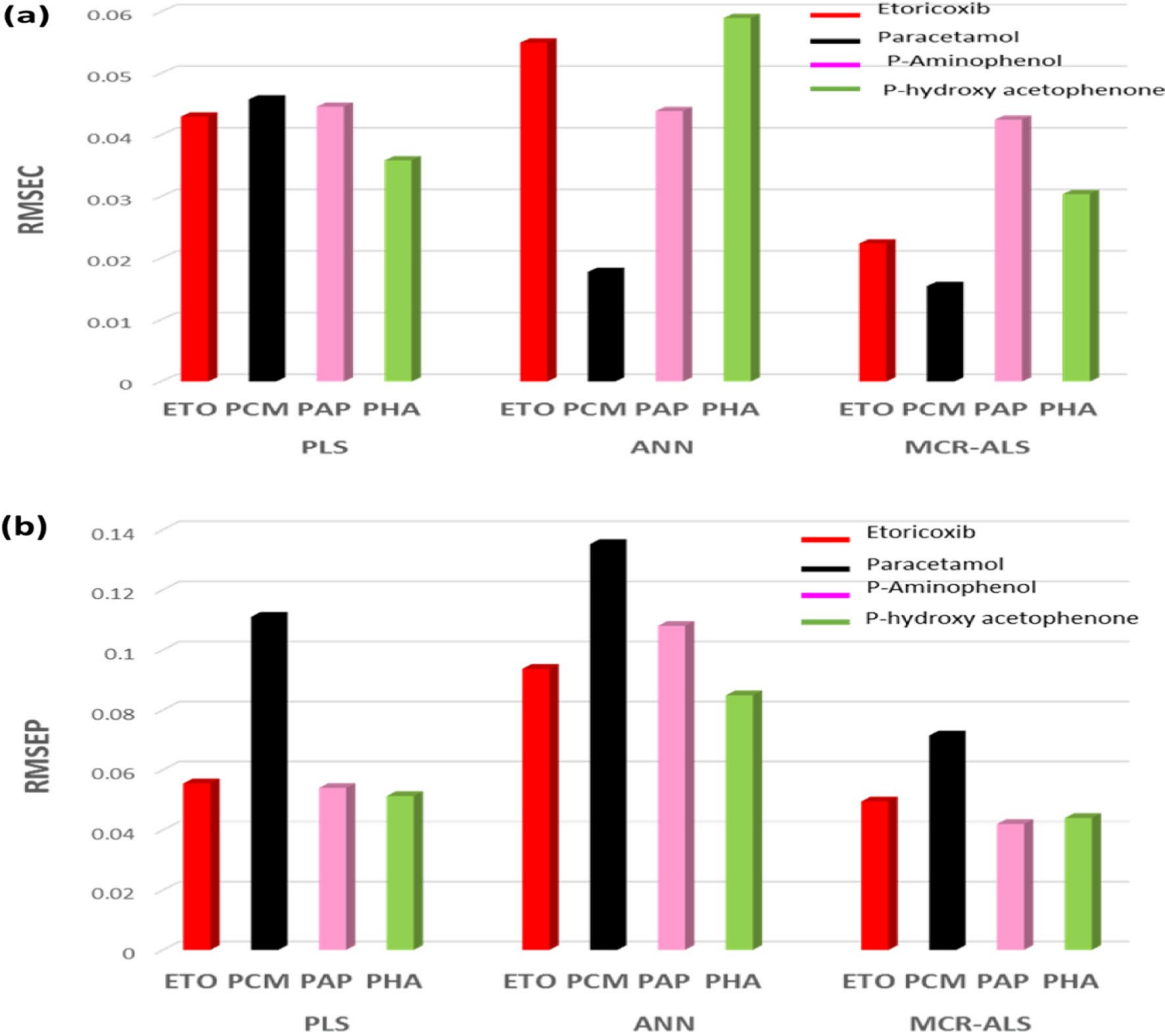
The calculated (**a**) RMSEC for each component achieved by the proposed calibration models and **b** RMSEP calculated by the corresponding validation model

The concentrations of four components in the validation set mixtures were determined using the developed models, the recovery %, RSD %, and root mean square error of prediction (RMSEP) were calculated with satisfactory results (Table 3). Column charts show the calculated RMSEC and RMSEP of the calibration and validation models for each component (Fig. 7). Finally, it was determined that MCR-ALS is the best model for quantitative analysis of the components because the findings showed that it has the lowest RMSEC and RMSEP.

**Table 3:** Prediction of validation set samples using the proposed chemometric models

Concentration (µg mL^−1^)				PLS				ANN				MCR-ALS			
				Recovery %				Recovery %				Recovery %			
ETO	PCM	PAP	PHA	ETO	PCM	PAP	PHA	ETO	PCM	PAP	PHA	ETO	PCM	PAP	PHA
3	10	3	5	99.76	98.83	98.92	99.18	98.59	98.02	98.81	99.12	100.68	99.47	98.77	98.91
7.5	4	5	5	101.01	99.08	99.54	98.27	99.28	100.20	99.12	98.90	98.44	100.35	99.98	99.45
3	8	5	4	99.98	100.44	100.88	98.66	98.22	98.43	100.57	99.97	100.26	99.11	98.49	99.62
6	8	4	6	100.06	98.91	99.73	100.94	99.72	98.50	98.62	100.37	100.67	99.76	98.88	100.06
4.5	10	5	6	99.25	100.18	101.45	100.39	99.50	98.83	100.56	101.60	99.22	99.90	101.30	100.02
6	10	2	2	99.99	99.76	102.62	98.48	100.90	99.44	99.04	98.45	99.86	100.70	98.14	98.83
3	6	2	3	100.82	99.11	101.81	98.56	99.67	99.43	98.69	98.30	100.33	98.77	99.65	98.92
Mean				100.12	99.49	100.71	99.21	99.41	98.98	99.34	99.53	99.92	99.72	99.32	99.40
RSD%				0.604	0.626	1.343	1.050	0.869	0.757	0.850	1.184	0.829	0.675	1.089	0.530
RMSEP^a^				0.0555	0.1110	0.0540	0.0513	0.0937	0.1353	0.1080	0.0849	0.0495	0.0715	0.0420	0.0440

^a^Root mean square error of prediction

### Assay of pharmaceutical formulations

Etoricoxib and PCM in Intacoxia-P tablet® tablets were successfully determined using the suggested chemometric models. The results showed excellent consistency with labeled concentrations. It was confirmed by good accuracy and a standard deviation of less than 2 that the pharmaceutical product’s excipients did not interfere the measurement of these drugs (Table 4).

**Table 4:** Quantitative determination of ETO and PCM in the dosage form by the proposed chemometric models

	Drug	PLS	ANN	MCR-ALS
Intacoxia-P tablet	Found % ± SD^a^			
	ETO	99.39 ± 1.070	98.14 ± 0.837	100.33 ± 1.039
	PCM	100.55 ± 0.817	100.84 ± 0.370	100.10 ± 1.016

^a^Average of three determinations of 3.6 µg mL^−1^ for ETO, and 10 µg mL^−1^ for PCM

### Statistical analysis

The results of the proposed chemometric models for determining ETO and PCM were statistically compared to those of the reported HPLC [31]. There was no significant difference between the proposed and reported method, judging by the calculated *t* and *F* values being lower than the tabulated ones (Table 5).

**Table 5:** Statistical comparison for the results obtained by the proposed chemometric models and the reported HPLC method for the determination of ETO and PCM in their pure powdered form

Parameter	PLS		ANN		MCR-ALS		Reported HPLC method^a^	
	ETO	PCM	ETO	PCM	ETO	PCM	ETO	PCM
Mean	100.21	99.49	99.13	98.98	99.92	99.42	99.98	99.89
S.D	0.605	0.626	0.746	0.749	0.828	0.645	0.992	1.102
Variance	0.366	0.391	0.556	0.562	0.685	0.416	0.984	1.214
n	7	7	7	7	7	7	9	9
Student t test^b^ (2.145)	0.327	0.854	1.884	1.868	0.593	0.998		
F value^b^ (4.15)	2.688	3.098	1.768	2.164	1.435	2.919		

^a^HPLC method using a C_18_ column as the stationary phase and a mixture consisting of (methanol: water) in ratio (70:30 v/v) as a mobile phase. The mobile phase was pumped at a flow rate of 1.0 mL min^−1^. UV detection was carried out at 235.0 nm

^b^The values in parentheses are the corresponding tabulated two-tailed values at* p* = *0.05*

## Conclusion

Multivariate calibration strategy based on a variety of chemometric models applied to a set of spectrum (all signal) is regarded as an effective alternative to the univariate calibration strategy relied on a set of datum (a single value corresponding to the maximum of UV–Vis spectrum) for complex mixture analysis. It has important applications and can extract important data from supplied datasets. In this study, an uncomplicated, accurate, easily accessible, and reasonably priced UV spectrophotometry was used to resolve the samples, interfering components, and severely overlapped spectra. Chemometrically aided UV spectrophotometric models, including PLS, ANN, and MCR-ALS, have been described with promising results for the quantification of ETO/PCM in their pharmaceutical dosage forms without any prior separation. Additionally, the suggested models succeeded in analyzing PCM impurities (PAP and PHA) quantitatively. The MCR-ALS was determined to be the most precise model. Additionally, it is the only model that can extract the spectrum profiles of the four components, so used for both quantitative and qualitative analysis.

## Data Availability

Datasets generated and/or analyzed during the current study are available from the corresponding author on reasonable request.
